# Relationship between proactive personality and career exploration among Chinese university students: mediating effects of career decision-making self-efficacy and career maturity

**DOI:** 10.3389/fpsyg.2026.1809829

**Published:** 2026-06-25

**Authors:** Zhaojun Meng, Kab Won Kang, Ke Wang

**Affiliations:** 1 Henan Normal University Henan Province Soft Science Research Base for Social Work and Social Governance, Xinxiang, China; 2 Henan Normal University, Xinxiang, China; 3 Graduate School of Education, Daejin University, Pocheon-si, Republic of Korea

**Keywords:** career decision-making self-efficacy (CDSE), career exploration (CEX), career maturity (CM), Chinese student, proactive personality (PP)

## Abstract

**Introduction:**

Given the intensifying employment difficulties faced by Chinese university students today, career exploration (CEX) has become increasingly essential. Examining its relationships with career decision-making self-efficacy (CDSE), career maturity (CM), and proactive personality (PP) may provide valuable insights for enhancing students' CEX.

**Methods:**

A quantitative research design was employed to examine the relationships among PP, CM, CDSE, and CEX among Chinese university students. Structural equation modeling was conducted while controlling for grade level.

**Results:**

The results showed that only PP and CDSE had significant positive effects on CEX. CDSE mediated the relationship between PP and CEX, whereas CM did not show a significant mediating effect.

**Discussion:**

The findings suggest that enhancing PP and CDSE may be beneficial for promoting Chinese university students' CEX and employability. Therefore, these factors should be considered in programs designed to improve students' CEX or career competence.

## 1 Introduction

As of 2025, the number of university graduates in China has reached an all-time high of 12.22 million (Ministry of Education of the People's Republic of China, 2024). In 2024, the employment rate among university graduates was 55.5%, falling below the 60% threshold (Zhaopin Limited, 2025), and employment competition is expected to intensify further in the coming years. Under these circumstances, university students may pursue occupations that are easier to obtain rather than those that match their aptitudes and values, which may lead to occupational maladjustment and repeated job changes. In addition, many university students select their majors or institutions without sufficient consideration of their interests or career values and therefore experience difficulties in career decision-making (Kim and Kim, 2012).

Career exploration (CEX) may help students better understand themselves and the world of work. The importance of CEX is consistent with the goals of the vocational guidance movement that emerged in the United States during the 1930s, which emphasized understanding personal interests, aptitudes, strengths, and weaknesses to identify suitable occupations (Crites, 1973). Such an approach remains relevant today (Yang, 2008), particularly in the context of increasing employment uncertainty among Chinese university students. To promote effective CEX, it is necessary to identify psychological factors associated with career-related attitudes and behaviors. Therefore, the present study focuses on proactive personality (PP), career decision-making self-efficacy (CDSE), and career maturity (CM) as key psychological constructs related to CEX among Chinese university students.

### 1.1 The relationship between PP and CEX

CEX is inherently self-initiated; therefore, individuals with stronger PP are more likely to engage effectively in CEX (Zikic and Hall, 2009). Proactive individuals actively gather career-related information and seek opportunities to improve their job-related skills (Qu et al., 2015; Fang et al., 2017; Green et al., 2020; Zhu et al., 2021). Previous studies have consistently reported positive relationships between PP and CEX (Qu et al., 2015), as well as between PP and career success (Seibert et al., 2001; Fang et al., 2017). PP was originally conceptualized by Bateman and Crant (1993) as a behavioral tendency to influence one's environment. It has also been defined as the tendency to identify opportunities, take initiative, overcome obstacles, and pursue change-oriented behaviors (Seibert et al., 1999; Ye, 2007).

### 1.2 The relationship between PP and CDSE

Individuals with stronger PP tend to exert greater control over their environment, approach tasks with a challenge-oriented mindset, and actively gather career-related information (Tian and Zheng, 2020; Ye, 2007; Tolentino et al., 2014; Qu et al., 2015; Fang et al., 2017). Accordingly, PP is expected to be positively associated with CDSE. Several empirical studies have supported this relationship (Duru and Söner, 2024; Qu et al., 2015; Li and Wang, 2016; Shang and Gan, 2009; Liang and Cheng, 2015; Fang et al., 2017). CDSE was introduced by Hackett and Betz (1981) as the application of self-efficacy theory to career development and later became a core construct of social cognitive career theory (Oh, 2014). CDSE generally refers to confidence in one's ability to successfully perform career decision-making tasks (Taylor and Betz, 1983; Betz et al., 1996; Nam et al., 2011).

### 1.3 The relationship between CM and CEX

CM refers to the development of career-related knowledge, attitudes, and skills (Park, 2007; Han and Lee, 2013; Kim and Kang, 1995). It also includes career problem-solving abilities and readiness for career decision-making (Jeong et al., 2012; Kim et al., 2015). Super (1955) conceptualized CM as a developmental process that progresses across life stages. Because CM involves career-related preparedness and competence, individuals with higher CM are expected to engage more actively in CEX. Previous studies have consistently demonstrated positive relationships between CM and CEX (Joo et al., 2013; Ko and Sim, 2014; Lee, 2006; Chung, 2014; Seo, 2016; Zhao et al., 2022). In the present study, CM includes career goals, professional confidence, professional value, professional autonomy, reliance on relatives and friends, and occupational reference.

### 1.4 The relationship between CDSE and CEX

Because CEX precedes career decision-making, individuals with higher CDSE are likely to perform more effectively in CEX activities. Several studies have reported positive associations between CDSE and CEX (Gushue et al., 2006; Lee et al., 2022; Qu et al., 2015; Seo, 2016; Zhao et al., 2022). Taylor and Betz (1983) identified five dimensions of CDSE: self-appraisal, occupational information, goal selection, planning, and problem solving.

### 1.5 The mediating roles of CDSE and CM between PP and CEX

Previous studies have shown that PP is positively related to both CDSE and CM (Hou et al., 2014; Yang and Chau, 2016; Fang et al., 2017). Individuals with stronger PP tend to actively shape their environment, gather career-related information, and overcome obstacles (Ye, 2007; Tian and Zheng, 2020; Tolentino et al., 2014). Based on these findings, PP is expected to positively influence CEX both directly and indirectly through CDSE and CM. However, to the best of our knowledge, no previous study has examined whether CDSE and CM mediate the relationship between PP and CEX while controlling for grade level.

### 1.6 Grade-level differences in PP, CDSE, CM, and CEX

Although CM generally increases with age (Super, 1955, 1981), previous studies have suggested that grade level may be more strongly associated with CM because educational systems influence career decision-making processes (Naidoo, 1998; Walsh and Osipow, 1973; Gottfredson, 1981). Research has also indicated that CM predicts CEX more effectively at higher grade levels (Kaminsky and Behrend, 2015). Because the present sample included relatively fewer upper-grade students, grade level was controlled to more accurately examine the relationships among the variables.

### 1.7 The context of Chinese employment culture

Chinese university students have grown up within a collectivistic social system that historically emphasized state-directed employment. From 1949 until approximately 1990, the Chinese government assigned jobs and workplaces to university graduates. Although this system gradually changed and was replaced by a market-oriented employment system by 2000, the historical legacy of state-assigned employment may still influence Chinese students' PP, CEX, CDSE, and CM. Accordingly, strengthening PP, CDSE, CM, and CEX may be particularly important for Chinese university students. Examining the relationships among these variables may therefore provide meaningful implications for the development of career education and career guidance programs aimed at improving university students' employability. The present study aims to examine the relationship between PP and CEX among Chinese university students, investigate the mediating roles of CDSE and CM, and explore the theoretical and practical implications of these relationships for career education and employability enhancement.

## 2 Methodology

### 2.1 Research subjects

Participants were recruited from four public universities in Henan Province, China. Data collection was conducted via mobile devices between 22 April and 6 May 2024. The final sample consisted of 295 students, including 59 men (20%) and 236 women (80%). By academic year, 118 students (40%) were in their first year, 77 (26.1%) were in their second year, 58 (19.7%) were in their third year, and 42 (14.2%) were in their fourth year.

### 2.2 Research ethics

According to the Measures for the Ethical Review of Life Science and Medical Research Involving Human Beings in China, studies using anonymized survey data that do not involve sensitive personal information may be exempt from formal ethical review. Because the present study used anonymous self-report questionnaire data and did not involve clinical intervention or sensitive personal information, formal ethical review was not required. However, this study was conducted with the approval of the Institutional Review Board (IRB) of Henan Normal University in Henan Province, China.

### 2.3 Measurement instrument

To achieve the purpose of this study, data on PP, CEX, CDSE, and CM were collected through self-report questionnaires, and the measurement instruments are presented in Table 1. Each item of all the measurement instruments was rated on a five-point Likert scale. PP was measured using the scale developed by Bateman and Crant (1993), which was translated into Chinese by Shang and Gan (2009) (see questionnaire in Appendix).

**Table 1:** Measurement instruments and reliability.

Instrument	Item numbers	Number of items	Cronbach's α	CR	AVE
PP	1–11	11	0.89	0.88	0.72
CDSE				0.97	0.88
Self-appraisal	1–6	6	0.87		
Information collection	7–15	9	0.88		
Goal selection	16–24	9	0.84		
Career planning	25–32	8	0.90		
Problem solving	33–39	7	0.87		
CM				0.84	0.57
Job goal setting	1, 4, 6^*^, 10, 12^*^, 22, 26, 31	8	0.84		
Career confidence	3^*^, 8^*^, 13^*^, 19^*^, 28^*^, 33^*^	6	0.84		
Job values	2^*^, 7^*^, 15^*^, 18^*^, 23^*^, 27^*^	6	0.68		
Career independence	14, 21, 25, 30	4	0.78		
Peer dependence	5^*^, 9^*^, 11^*^, 32^*^	4	0.69		
Job reference	16, 17^*^, 20, 24, 29^*^, 34	6	0.51		
CEX				0.89	0.73
Environmental Exploration	1–5	5	0.84		
Self-exploration	6, 8–11	5	0.75		
Goal exploration	7, 12–14	4	0.80		
Information exploration	15–18	4	0.82		

^*^Reverse scoring items. CDSE, career decision-making self-efficacy; CEX, career exploration; CM, career maturity; PP, proactive personality.

CM was assessed using the scale originally developed by Lee (2001), which was translated into Chinese by Zhang et al. (2006). This instrument includes 34 items across six subscales: job goal setting (eight items), career confidence (six items), job values (six items), career independence (four items), peer dependence (four items), and job reference (six items) (see questionnaire in Appendix).

CDSE was measured using the scale developed by Taylor and Betz (1983), which was translated into Chinese by Peng and Long (2001). This scale comprises 39 items distributed across five subscales: self-appraisal (six items), occupational information collection (nine items), goal selection (nine items), career planning (eight items), and problem-solving (seven items; see questionnaire in Appendix). The Career Exploration Survey, originally developed by Stumpf et al. (1983), and later translated and adapted into Chinese by Xu (2008), was used in this study. The original instrument consists of 55 items across 16 subscales. For the present study, 18 items were selected and reorganized based on Xu's (2008) version. The selected items were renumbered consecutively for clarity in the questionnaire (see Appendix). The final CEX scale comprised four subdimensions: environmental exploration (five items), self-exploration (five items), goal exploration (five items), and information exploration (five items; see Table 1).

### 2.4 Data processing

There were no missing values in the dataset, and the data analysis was conducted using SPSS 26 and AMOS 26. Pearson's correlation coefficients were calculated, a structural equation model (SEM) was tested, and the maximum likelihood (ML) method was used to perform a parameter estimation. To assess the mediating effects, the bootstrap resampling technique was employed with 2,000 iterations. Statistical significance was tested using the bias-corrected percentile method, with a 95% confidence interval, and in addition, the statistical significance of differences in the magnitude of standardized path coefficients was examined.

Given that all four measurement instruments utilized a five-point Likert scale, the possibility of common method bias (CMB) was considered. To assess this, a common method factor was incorporated into the measurement model. Specifically, a comparison was made between the χ^2^ value of the model with the common method factor and the χ^2^ value of the model without it. A statistically significant difference in χ^2^ indicates the presence of common method bias (Collier, 2020).

In the present study, the χ^2^ value of the measurement model with the common method factor was 244.050 (*df* = 83), whereas the χ^2^ value of the model without the common method factor was 241.562 (*df* = 84), a difference in χ^2^ of 2.788, with 1 degree of freedom. Given that the critical value of χ^2^ at *df* = 1 and α = 0.05 is 3.84, the observed difference did not exceed this threshold. Therefore, it was concluded that common method bias was not a significant concern in this study.

### 2.5 Verification of the measurement model

#### 2.5.1 Convergent validity

To reduce the number of observed variables and improve model parsimony, item parceling was conducted using a random assignment procedure. The PP, CEX, CM, and CDSE scales were divided into three, three, four, and five parcels, respectively.

#### 2.5.2 Model fit

In the present study, the measurement model demonstrated acceptable fit with the following indices: Tucker–Lewis index (TLI) = 0.950; root mean square residual (RMR) = 0.017; standardized root mean square residual (SRMR) = 0.046; and root mean square error of approximation (RMSEA) = 0.080.

#### 2.5.3 Discriminant validity

Table 2 presents the results of the discriminant validity analysis. Discriminant validity was evaluated using Fornell and Larcker's (1981) criterion, whereby the square root of the AVE for each latent construct should exceed the squared correlations between that construct and any other latent construct in the model. Since the AVE values (Bold font) for all four constructs exceeded the squared correlations with other latent variables, the results confirm that each construct demonstrates adequate discriminant validity, indicating that all scales measure distinct theoretical concepts.

**Table 2:** Discriminant of measurement instrument.

Variables	PP	CM	CDSE	CEX
PP	0.**72**			
CM	0.19	0.**57**		
CDSE	0.48	0.34	0.**88**	
CEX	0.48	0.23	0.50	0.**73**

CDSE, career decision-making self-efficacy; CEX, career exploration; CM, career maturity; PP, proactive personality. Bold values indicate AVE values.

To assess the normality of the observed variables within the measurement model, skewness and kurtosis statistics were examined. The results indicated that the absolute values of skewness ranged from 0.05 to 0.48, remaining well below the commonly accepted threshold of ±2.0, thereby suggesting no significant skew in the distribution. Similarly, the absolute values of kurtosis ranged from 0.03 to 0.80, which is also within the acceptable range of ±4 as applied in SPSS (a more conservative criterion than the general threshold of ±7). To examine the linearity among the indicator variables, Pearson's correlation coefficients were computed (see Table 3). The results indicated that the correlation coefficients ranged from *r* = 0.22 to *r* = 0.89, all of which were statistically significant at the *p* < 0.01 level. This suggests that the variables exhibited a significant linear relationship.

**Table 3:** Correlation coefficient between measurements (*n* = 295).

Variables	PP1	PP2	PP3	CEX1	CEX2	CEX3	CM1	CM2	CM3	CM4	CDSE1	CDSE2	CDSE3	CDSE4
PP1														
PP2	0.676^**^													
PP3	0.747^**^	0.721^**^												
CEX1	0.466^**^	0.415^**^	0.478^**^											
CEX2	0.560^**^	0.561^**^	0.597^**^	0.739^**^										
CEX3	0.420^**^	0.395^**^	0.415^**^	0.717^**^	0.739^**^									
CM1	0.271^**^	0.204^**^	0.262^**^	0.221^**^	0.281^**^	0.260^**^								
CM2	0.359^**^	0.317^**^	0.358^**^	0.279^**^	0.415^**^	0.381^**^	0.709^**^							
CM3	0.253^**^	0.226^**^	0.295^**^	0.217^**^	0.293^**^	0.244^**^	0.556^**^	0.582^**^						
CM4	0.284^**^	0.223^**^	0.267^**^	0.323^**^	0.417^**^	0.461^**^	0.475^**^	0.533^**^	0.563^**^					
CDSE1	0.558^**^	0.557^**^	0.518^**^	0.488^**^	0.601^**^	0.559^**^	0.361^**^	0.478^**^	0.276^**^	0.408^**^				
CDSE2	0.572^**^	0.562^**^	0.521^**^	0.545^**^	0.604^**^	0.572^**^	0.354^**^	0.464^**^	0.349^**^	0.466^**^	0.851^**^			
CDSE3	0.576^**^	0.535^**^	0.538^**^	0.539^**^	0.612^**^	0.568^**^	0.419^**^	0.540^**^	0.373^**^	0.463^**^	0.872^**^	0.809^**^		
CDSE4	0.571^**^	0.561^**^	0.545^**^	0.526^**^	0.612^**^	0.535^**^	0.404^**^	0.496^**^	0.367^**^	0.437^**^	0.873^**^	0.872^**^	0.854^**^	
CDSE5	0.571^**^	0.544^**^	0.482^**^	0.521^**^	0.592^**^	0.571^**^	0.311^**^	0.445^**^	0.341^**^	0.429^**^	0.864^**^	0.887^**^	0.811^**^	0.855^**^

^**^P < 0.01.

PP1 to PP3 are the indicators of PP latent variable.

CEX1 to CEX3 are indicators of CEX latent variable.

CM1 to CM4 are indicators of CM latent variable.

CFSE1 to CDSE44 are indicators of CDSE latent variable.

## 3 Results

### 3.1 Descriptive statistics and correlation coefficients

The mean scores of the four latent variables, converted to a five-point scale, were as follows: PP had a mean of 3.37 (SD = 0.69), CM had a mean of 3.25 (SD = 0.40), CDSE had a mean of 3.27 (SD = 0.65), and CEX had a mean of 3.21 (SD = 0.53). The Pearson's product correlation coefficients among the latent variables are presented in Table 4. The correlation coefficients ranged from *r* = 0.44 to *r* = 0.71, all of which were statistically significant at the *p* < 0.001 level, indicating moderate to strong positive associations among the variables.

**Table 4:** Correlation coefficient between latent variables (*n* = 295).

Variables	PP	CM	CDSE
CM	0.44^**^		
CDSE	0.69^**^	0.58^**^	
CEX	0.69^**^	0.48^**^	0.71^**^

CDSE, career decision-making self-efficacy; CEX, career exploration; CM, career maturity; PP, proactive personality.

^**^P < 0.01.

### 3.2 Relationships among latent variables

**Figure 1:**
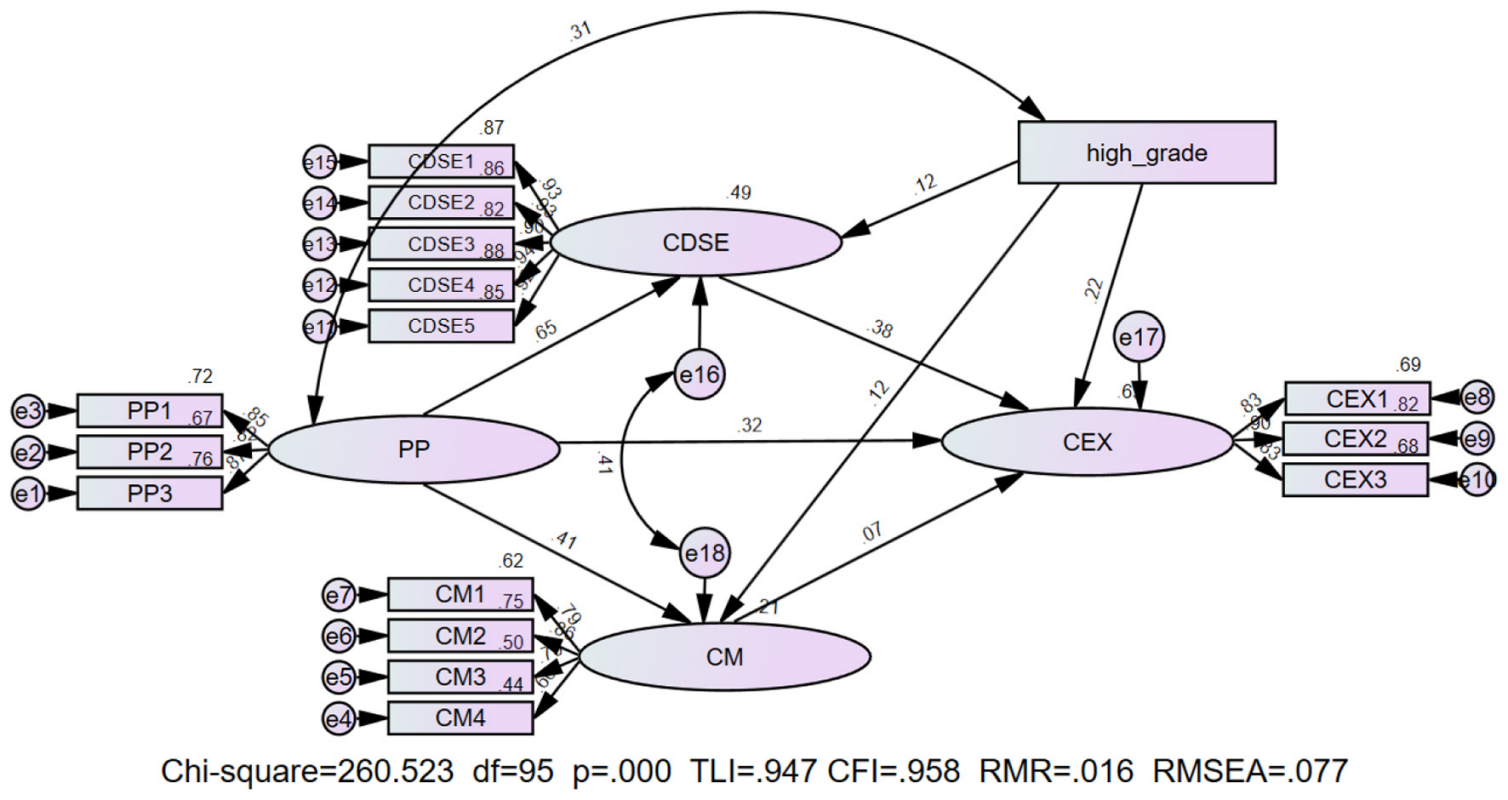
Structural model. PP, proactive personality; CM, career maturity; CDSE, career decision-making self-efficacy; CEX, career exploration.

SEM was conducted to examine the relationships between the PP and CEX latent variables and to determine whether CDSE and CM mediate the relationship between PP and CEX. In this model, participants' academic grade level was included as a control variable. The first- or second-year students were designated as the reference group, while third-year and fourth-year students were grouped as the comparison group (referred to as the “high grade”). It was assumed that the comparison group would exhibit higher levels of CDSE, CM, and CEX. The control variable “higher grade” was coded as a dummy variable, where students in the first and second years were coded as 0, and those in the third and fourth years were coded as 1. The structural model is illustrated in Figure 1. The model's goodness-of-fit indices were as follows: TLI = 0.947, RMR = 0.016, SRMR = 0.045, and RMSEA = 0.077. Statistical significance was assessed using the bias-corrected confidence interval method.

### 3.3 Direct effects

Table 5 presents the results of the path coefficient analysis for the structural model. All path coefficients between latent variables were estimated while controlling for the effect of academic grade level. The path from PP to CEX was statistically significant (β = 0.32), and PP was significantly positively related to CDSE (β = 0.66) and CM (β = 0.41). Furthermore, although CDSE significantly predicted CEX (β = 0.38), CM did not significantly predict CEX (β = 0.07, *p* > 0.05). However, the mediating effect of CDSE on the relationship between PP and CEX was significant (indirect effect β = 0.17), indicating that CDSE serves as a positive mediator that enhances the relationship between PP and CEX, increasing the total effect from β = 0.32 to β = 0.49 (*p* < 0.001). In contrast, the mediating effect of CM in the PP–CEX relationship was positive but not statistically significant (indirect effect β = 0.02, *p* > 0.05).

**Table 5:** Path coefficients between latent variables.

Path	β	95% CI (β)	Ratio of explanation
PP (1) → CEX	0.32^***^	0.19 – 0.46	
PP → CDSE	0.65^***^	0.57 – 0.75	
PP → CM	0.41^***^	0.29 – 0.53	
CDSE (2) → CEX	0.38^***^	0.23 – 0.53	
CM → CEX	0.07	−0.04 – 0.18	
High_grade → CEX	0.22^***^	0.18 – 0.37	
High_grade → CDSE	0.12^**^	0.01 – 0.23	
High_grade → CM	0.12^*^	0.01 – 0.24	
PP → CDSE → CEX (3)	0.17^***^	0.11 – 0.29	
PP → CM → CEX (4)	0.02	−0.01 – 0.06	24.67% (1 + 2 + 3)
Comparison of mediation effects (3–4)	0.15^**^	0.07 – 0.25	

CDSE, career decision-making self-efficacy; CEX, career exploration; CM, career maturity; PP, proactive personality.

^*^P < 0.05, ^**^P < 0.01, and ^***^P < 0.001.

### 3.4 Indirect effects

The mediating effect of CDSE between PP and CEX was found to be significantly greater than that between CM and CEX (β = 0.15, *p* < 0.01). However, the combined direct effect of PP and CDSE on CEX, along with the indirect effect mediated by CDSE, accounted for approximately 25% of the variance. The third-year and fourth-year student groups exhibited significantly higher levels of CEX, CDSE, and CM compared to the first-year and second-year student groups. Although a correlation between CM and CEX exists, the covariance linkage between these two variables in the model suppressed the effect of CM on CEX.

## 4 Discussion

### 4.1 Discussion of research results

The results of this study provide several important insights. First, the levels of PP, CDSE, CM, and CEX among Chinese university students were slightly above the midpoint (3.0) on a five-point scale, indicating room for improvement. Among these, enhancing PP is particularly critical. Given China's educational and sociocultural context, conformity and collective adaptation may traditionally have been emphasized more than individual initiative and proactive behavior. However, as university students are now expected to actively navigate their own career paths and employment, efforts to strengthen PPs are essential. As a first step, university administrators should consider developing and offering programs that promote proactive traits among students.

Second, the results reveal that PP, CDSE, and CM were all positively associated with CEX. These findings are consistent with a substantial body of empirical research on the relationships between PP and CEX (Qu et al., 2015; Zhu et al., 2021), PP and CM (Fang et al., 2017), PP and CDSE (Duru and Söner, 2024; Qu et al., 2015; Li and Wang, 2016; Shang and Gan, 2009; Liang and Cheng, 2015; Fang et al., 2017), CDSE and CEX (Lee et al., 2022; Wang and Chen, 2014; Liu and Liu, 2014; Gushue et al., 2006; Joo et al., 2013; Seo, 2016), and CM and CEX (Joo et al., 2013; Hwang and Moon, 2012; Ko and Sim, 2014; Lee, 2006; Chung, 2014; Seo, 2016; Zhao et al., 2022). In the correlation analysis, CM and CDSE were associated with CEX (*r* = 0.71 and *r* = 0.48), suggesting that CDSE exhibited a relatively stronger correlation. However, in the structural model, owing to overlapping variance, the effect of CM was not statistically significant. This may be due to the suppression of the influence of CM by the shared variance with CDSE. Consequently, from a structural model perspective, increasing PP and CDSE may play a more central role in promoting CEX. Therefore, interventions or programs aiming to enhance CEX should prioritize PP and CDSE over CM. PP emerged as a particularly influential variable, as it influences both CDSE and CEX.

Third, structural equation modeling indicates that PP had a direct positive effect on CEX (β = 0.32) and that CDSE had a similarly strong effect (β = 0.38). These results suggest that CDSE is nearly as critical as PP in influencing CEX. In this study, PP influenced CEX not only directly but also indirectly through CDSE. This mediating effect aligns with previous findings (Lent and Brown, 2013). Moreover, these two variables together accounted for approximately 25% of the variance in CEX, which represents a meaningful proportion and reinforces their importance in career development interventions.

Fourth, the results reveal that students in higher academic years reported significantly higher levels of PP, CDSE, and CM. This suggests the importance of early intervention. For example, efforts to enhance PP and CDSE should be initiated during the early stages of university education to foster career exploration and improve employment outcomes.

Fifth, while it is possible that the levels of the studied variables differ by gender, the effect of gender was not controlled for in the current structural model. Since approximately 80% of the participants were women, future research should either control for gender or examine its moderating role to improve the generalizability and stability of the findings.

Sixth, the present study controlled for grade level when examining the relationships among PP, CDSE, CM, and CEX. Previous studies have rarely considered grade level as a control variable despite evidence that career-related variables may differ across academic years. Even after controlling for grade level, the relationships among the major variables remained consistent with previous findings, suggesting that the associations between PP, CDSE, and CEX are relatively robust. The results of the present study indicate that, even after controlling for the effect of grade level on the relationships among these variables, the findings show trends similar to those reported in prior research. This suggests that, at least for the relationships between PP and CEX and between CDSE and CEX, the positive associations are more robust.

### 4.2 Limitations and suggestions

This study has several limitations. First, the sample consisted only of students from four universities located in Henan Province, China. Therefore, generalizing the results to the broader population of Chinese university students should be done with caution, particularly when interpreting the mean levels of the variables. Future research should improve sample representativeness by including participants from diverse regions and institutions across China and by employing more nationally representative samples.

Second, the data used in this study were hierarchical in nature because participants were nested within universities. However, multilevel modeling techniques were not applied, and the structural equation model did not account for the nested structure of the data. As a result, potential institutional differences could not be adequately examined. Future studies should apply multilevel modeling approaches to better account for institutional and contextual effects.

Third, gender should be treated as a control variable in future studies. Approximately 80% of the participants in the present study were female, and previous research has suggested that CEX may vary by gender (e.g., Zikic and Hall, 2009). In addition, PP and career-related behaviors may differ across gender groups in certain cultural and social contexts. Therefore, the gender imbalance in the present sample may have reduced the external validity and generalizability of the findings. Future research should employ more gender-balanced samples to improve the robustness of the results.

Fourth, the present study employed a cross-sectional design, which limits the ability to infer causal relationships or developmental changes over time. Future research should use longitudinal designs to examine the temporal relationships among PP, CDSE, CM, and CEX.

Fifth, CEX was measured primarily through cognitive and attitudinal dimensions rather than through concrete behavioral indicators. Future studies should incorporate behavioral measures such as internship participation, job-search activities, career preparation behaviors, and employment-related outcomes to better examine the practical implications of CEX.

## 5 Conclusion and implications

On the basis of the results and discussion of this study, several conclusions can be drawn. As part of broader efforts to address the employment difficulties faced by Chinese university students, it is necessary to strengthen levels of PP, CDSE, and CEX. These variables are closely related to career preparation and require initiative and self-directed behavior. Such characteristics may be less emphasized in educational and social environments that traditionally value conformity and collective adaptation. Therefore, universities should consider developing and implementing programs aimed at enhancing students' PP, CDSE, and CEX.

To enhance students' CEX, focusing on PP and CDSE may reduce the need for separate interventions specifically targeting CM. These findings appear to apply regardless of grade level. In particular, PP emerged as a particularly influential variable because it positively affected both CDSE and CEX. CDSE was also important because it showed a strong positive relationship with CEX and significantly mediated the relationship between PP and CEX. Compared with upper-grade students, lower-grade students may exhibit lower levels of PP, CDSE, CM, and CEX. Therefore, interventions aimed at strengthening these variables may be particularly important during the early years of university education.

Although CM was positively correlated with CEX, it did not significantly predict CEX in the structural model. This finding may be explained by the relatively stronger association between CDSE and CEX, as well as the shared variance between CM and CDSE. These results suggest that when designing educational interventions intended to enhance specific career-related behaviors such as CEX, constructs with lower levels of generality may be more practically effective. To ensure that CEX among Chinese university students leads to stronger employability, the development of PP should be considered a fundamental priority.

## Data Availability

The raw data supporting the conclusions of this article will be made available by the authors, without undue reservation.
