# Supplementary material for: Relationship between proactive personality and career exploration among Chinese university students: mediating effects of career decision-making self-efficacy and career maturity
Source: Front Psychol. 2026 Jun 25;17:1809829. doi: 10.3389/fpsyg.2026.1809829 (PMC13345841; doi:10.3389/fpsyg.2026.1809829)
Supplement: Supplementary file 1 [file Supplementary_file_1.docx]

Appendix

Questionnaire

Hello students,

This survey is designed to explore the relationships among several psychological characteristics. Your honest responses are very important for this study. It is expected to take approximately 5 minutes to complete. Since participation in this survey is voluntary, you may choose not to participate. All responses will remain confidential and will be used solely for research purposes. Thank you.

1. Do you agree to participate in this survey?

a) Yes b) No

2. What is your gender?

a) Male b) Female

3. What year are you in?

a) 1st year b) 2nd year c) 3rd year d) 4th year

Please read each of the following statements and choose the number that best reflects how well it applies to you.

① Strongly disagree (1 point) ② Disagree (2 points) ③ Neutral (3 points)

④ Agree (4 points) ⑤ Strongly agree (5 points)

Proactive Personality

1. If I see someone in difficulty, I will do everything I can to help.

2. I am good at turning problems into opportunities.

3. I am constantly looking for better ways to do things.

4. When I encounter problems, I face them head-on.

5. I like to challenge the status quo.

6. If I believe in an idea, no obstacle can stop me from pursuing it.

7. If I firmly believe in something, I will pursue it regardless of the chances of success or failure.

8. There is nothing more exciting than seeing my ideas become reality.

9. I am always looking for new ways to improve my life.

10. I enjoy the challenge of confronting and overcoming obstacles to my ideas.

11. I always want to feel that I am special in a group, perhaps even in the world.

Career Exploration

1. I understand the various possible directions for employment.

2. I obtain information about specific positions or companies.

3. I proactively seek information from knowledgeable people in the career fields in which I am interested.

4. I gather a wide range of employment information, including general job opportunities in fields that interest me.

5. I seek information about the career fields that interest me.

6. I pay attention to my personal traits such as personality, interests, and abilities.

7. I participate in various career counseling activities to clarify my career goals.

8. I think about what kind of job suits me best.

9. I reflect on my past experiences.

10. I deeply consider the direction of my future career path.

11. I think about how to integrate my past experiences into my future career development.

12. I engage in various occupational activities to gain experience.

13. I look for opportunities to demonstrate my skills.

14. I try different specific job roles and assess whether I enjoy them.

15. I understand the main responsibilities of the career field in which I am interested.

16. I am knowledgeable about job positions, organizations, and the current job market.

17. I understand my career options in terms of personal interests, abilities, and career direction.

18. I know how to plan the path of my future career.

Career Maturity

1. I have already decided what kind of job I want in the future.

2. When choosing a career, money is the most important thing; nothing else matters.

3. I’m worried that I won’t be able to find the job I want.

4. Although I’m still a student, I can imagine what my future job will be like.

5. If my parents disapprove of the career I want, I would feel conflicted about choosing it.

6. I am interested in many careers, so I find it difficult to choose just one.

7. I would choose a career that others admire over one that truly suits me.

8. I am not certain whether I can succeed in the career I have chosen.

9. I will choose my career based on my parents’ expectations.

10. Since I have already chosen a career, I no longer need to worry about my career decisions.

11. I will choose a career based on my friends’ wishes.

12. I often think about possible careers, but I have not yet settled on a specific goal.

13. Because I do not have much confidence in myself, I doubt that succeeding in my chosen career would help me become the person that I want to be.

14. No matter what others say, I think I will choose a career I like.

15. As long as I can make a lot of money, I do not care what kind of job I have.

16. When reading the news, I enjoy articles about people who have succeeded in fields in which I am interested.

17. Other people’s support is extremely important to me when choosing a career.

18. Regardless of my interests, I want to choose a prestigious career.

19. I worry that the career I choose will not help me become the person I want to be.

20. I want to interact with people who work in the career fields in which I am interested.

21. I will choose my career based on my own standards.

22. I have made a clear decision about my career.

23. When choosing a job, I will prioritize opportunities for promotion over the nature of the work.

24. I enjoy talking with alumni about academics and future careers.

25. To live my own life, I will choose a career based on my own beliefs.

26. Although I might change my mind later, I have chosen a career that appeals to me.

27. I prioritize high-paying jobs over those that provide personal fulfillment.

28. I lack confidence in myself and my abilities.

29. When I see people succeed in the career they are passionate about, I tend to imitate them to become like them.

30. No matter what others say, I need to choose a career that matches my interests.

31. I have not yet made a clear decision about my career.

32. I feel that if I choose a career that my parents disapprove of, I will regret it in the future.

33. No matter how hard I try, I may not be able to get the job I want.

34. I hope to receive counseling to better understand my interests and personality traits.

Self-efficacy in Career Making Decisions

1. I can list several careers or jobs that interest me.

2. I search for information about the careers or jobs in which I am interested.

3. I can choose a career or job that is suitable for my personal future.

4. I have developed short-term and long-term plans for my career and job goals.

5. Even when I feel discouraged, I persist in working toward my career goals.

6. I have identified my ideal career or job.

7. I seek information about employers that hire graduates from my academic field.

8. I select one career or job from the possible options I am currently considering.

9. I can identify the action steps I need to take to successfully obtain the career or job I have chosen.

10. I evaluate what I value most in a given career or job.

11. I understand the future outlook of a certain career or job.

12. I am able to choose a career or job that fits the lifestyle I desire.

13. When making a career decision, I do not worry about whether it is right or wrong.

14. I obtain recommendation letters from teachers and counsellors for job applications.

15. I can manage financial difficulties that arise during my job search.

16. I have identified the career or job I am most competent in.

17. I ask my teachers about careers and jobs related to my major.

18. I can choose a career or job even if my parents do not approve of it.

19. I gain work experience related to my future career or job goals.

20. When my parents or friends expect me to pursue a career beyond my abilities, I go against their wishes.

21. I can describe the job duties of the career I want to pursue.

22. I find and attend job fairs through talent exchange centers.

23. I handle various conflicts with a boyfriend or girlfriend that arise when I am job searching.

24. To achieve my career goals, I can list what I am willing and not willing to give up.

25. I research current and future employment trends for certain careers.

26. I can choose a career or job that matches my interests.

27. I will decide whether to pursue graduate school or vocational training on the basis of my career goals.

28. I investigate the average monthly or annual income for a specific career or job.

29. I can choose a career or job that matches my abilities.

30. I learn skills outside my major that are helpful for my future career.

31. I can accurately assess my own abilities.

32. I can talk with someone who is currently working in a career or job that interests me.

33. I can choose the best career or job for me, even if it requires extra effort.

34. I use various social connections to obtain career and job information.

35. I use national employment policies and regulations to protect my legitimate rights.

36. I search for information about graduate school admissions.

37. I choose the career or job I want, even if employment opportunities in that field are declining.

38. I can successfully handle the job interview process.

39. When job opportunities are severely limited, I can develop temporary coping strategies.
